# Spatial Distribution of the Micro-Mechanical Properties in High-Translucent CAD/CAM Resin-Composite Blocks

**DOI:** 10.3390/ma13153352

**Published:** 2020-07-28

**Authors:** Nicoleta Ilie

**Affiliations:** Department of Conservative Dentistry and Periodontology, University Hospital, LMU Munich, Goethestr. 70, D-80336 Munich, Germany; nilie@dent.med.uni-muenchen.de; Tel.: +49-89-44005-9412; Fax: +49-89-44005-9302

**Keywords:** CAD/CAM resin-based composites, micro-mechanical properties, Vickers hardness, Martens hardness, indentation modulus, creep, elastic and total indentation work

## Abstract

Industrially cured, high-translucent computer-aided design/computer-aided manufacturing (CAD/CAM) resin-based composites (RBC) are the most recently launched dental restoratives. Clinical treatments and laboratory tests are based on a homogeneous distribution of properties within CAD/CAM blocks to obtain constant and reproducible results. The study therefore aims to determine the spatial distribution of various micro-mechanical parameters (Vickers hardness, Martens hardness, indentation modulus, creep, elastic and total indentation work) in five representative CAD/CAM RBCs. The properties of the tooth structure were evaluated under similar conditions. Filler size and shape were analyzed by scanning electron microscopy. A multivariate analysis (general linear model) identified a very strong influence of the material on all measured properties (*p* < 0.001; partial eta squared η_P_^2^ > 0.943), whereby the most sensitive parameters when identifying differences within regions were the indentation modulus and the elastic indentation work. CAD/CAM RBC blocks show gradually varying properties that can increase or decrease from central to peripheral areas regardless of the chemical composition of the materials or the inorganic filler fraction. The degree of variation in the measured properties is material-specific and less than 8.7%. Clinical applications and in vitro study designs should consider slight inhomogeneity in CAD/CAM RBC blocks, while the location of the regions with best mechanical performance depends on the material.

## 1. Introduction

Resin-based composites (RBC) are quoted as the most suitable restorative materials to meet the aesthetic and functional requirements of modern times [1]. They enable the shade [2,3] and translucency [4] to be perfectly matched to the natural teeth, are suitable for a minimal invasive treatment, and offer good mechanical properties that are comparable to those of the human dentin [5]. In light-cured RBC restorations, however, these properties are directly related to the quality of curing, and in consequence to the amount of radiant exposure that reaches the surface of a restoration [6]. The latter is a cumulus of events relating to the quality, type and design of the light curing unit, the tooth anatomy, the type and depth of a restoration, as well as to the operator handling errors [7]. The quality of curing can be affected even more in deeper and peripheral areas of an RBC restoration, considering that the light is attenuated while passing through the material during curing, which induces a gradient of the properties within the restoration [8]. The posterior region represents the greatest mechanical challenge in dental RBC restorations. One factor that contributes to this challenge is the polymerization shrinkage and the associated tension, which can adversely affect the bond between the RBC and tooth [9] and thus the longevity of the restoration. Nonetheless, in long-term clinical studies (up to 30 years), light-cured RBCs placed in the posterior region are rated as excellent restorative materials for small to medium-sized defects [10,11]. In comparison to amalgam restorations, posterior RBCs restorations have survival rates that are equivalent or even higher after 12 [12] or even 19 years [13]. A comprehensive analysis of the clinical studies published in the period 1995–2005 and 2006–2016 shows interesting trends in the clinical behavior of RBC restorations [14]. The survival rates of RBC posterior restorations in the two decades are indicated as an average of 89.41% and 86.87%, which shows little difference. However, the reasons for the failure of RBC restorations have changed significantly. Secondary caries (29.47%) and RBC fractures (28.84%) were the main causes of failure in the 1995–2005 decade, while tooth fractures (3.45%) were rarely observed. In comparison, the RBC fracture (39.07%) proved to be the main reason for the failure of the RBC posterior restorations in the later decade (2006–2016), followed by secondary caries (25.68%) and tooth fracture (23.76%). The analysis clearly shows a significantly increased incidence of RBC fractures in recent years [14]. While this is said to be related to the increased use of RBCs in larger restorations and possible changes in material properties [14], the interface between RBC and tooth structure as a weak link in a restoration could also be considered a cause of RBC restorations failure.

An attempt to improve and homogenize the mechanical properties in RBCs, especially when used in large restorations, is their industrial curing in blocks from which a dental restoration is obtained by subtractive manufacturing via computer-aided design/computer-aided manufacturing (CAD/CAM)-machining. The resin matrix is similar in industrially cured CAD/CAM and light-cured RBCs, and consists of polymerized difunctional methacrylate monomers, while the most frequently used are the basis monomers Bis-GMA (Bisphenol A-diglycidyl dimethacrylate), Bis-EMA (ethoxylated bisphenol A dimethacrylate), UDMA (urethane dimethacrylate), and TEGDMA (Triethylene glycol dimethacrylate) [5,15]. The inorganic filler system is also comparable in both RBC categories, and consists of different mixtures of crystalline (oxides: e.g., SiO_2_, ZrO_2_) or amorphous (glasses: e.g., BaO-Al_2_O_3_-SiO_2_- glass, SiO_2_-glass) fillers of nano to micrometer size, with various filler size distribution (uni- to multimodal), and filler morphology (e.g., isolated nano-filler, cluster, splitter, whiskers, fiber) [5,15]. In contrast to the in situ polymerization of light-curing RBCs, the CAD/CAM RBCs underwent thermal curing and controlled isostatic pressure during polymerization. These curing conditions produce improved physical properties compared to the incrementally placed light-cured RBCs [16], that are attributed to an increased degree of monomer conversion and network density [17], and presumably also to an improved filler-matrix interaction [18]. Reduced air inclusions owed to isostatic pressure is also reported [16] which may have a positive effect on the mechanical stability of a restoration. Other encouraging effects are the reduced amount of monomer release, and the reduced toxicity [16]. There is also evidence that CAD/CAM RBCs yield lower Streptococcus mutans and plaque biofilm formation compared to light-cured RBCs [19], which are important features in reducing secondary caries formation.

The high-translucent CAD/CAM RBCs are the most recently launched dental restoratives. This material category focuses on improved aesthetics, while the increased translucency enables the use of not only dual-cured RBCs but also pure light-cured RBCs for luting. A CAD/CAM restoration for clinical use is randomly milled out of a CAD/CAM block without paying attention to the exact position in the block. The same applies to laboratory tests, since many test specimens can be fabricated from a single CAD/CAM block. As a result, clinical treatments and laboratory tests are based on a homogeneous distribution of properties within a CAD/CAM block in order to obtain constant and reproducible results. Manufacturers are promoting the homogeneity of properties in CAD/CAM blocks, but this has not yet been checked.

The aim of the present study was therefore to evaluate the homogeneity of the micro-mechanical properties in highly translucent CAD/CAM RBC blocks, taking into account the spatial distribution of a battery of depth-sensing indentation parameters.

The null hypotheses tested were: (a) The spatial distribution of the analyzed properties is similar in all CAD/CAM blocks, and (b) Parameters that were determined at different locations are similar within a CAD/CAM block.

## 2. Materials and Methods

### 2.1. Specimen Preparation

The spatial distribution of the micro-mechanical properties of CAD-CAD RBC blocks was monitored by a depth-sensing indentation test. To this purpose five representative high-translucent CAD/CAM RBCs were selected (Table 1).

**Figure 1:**
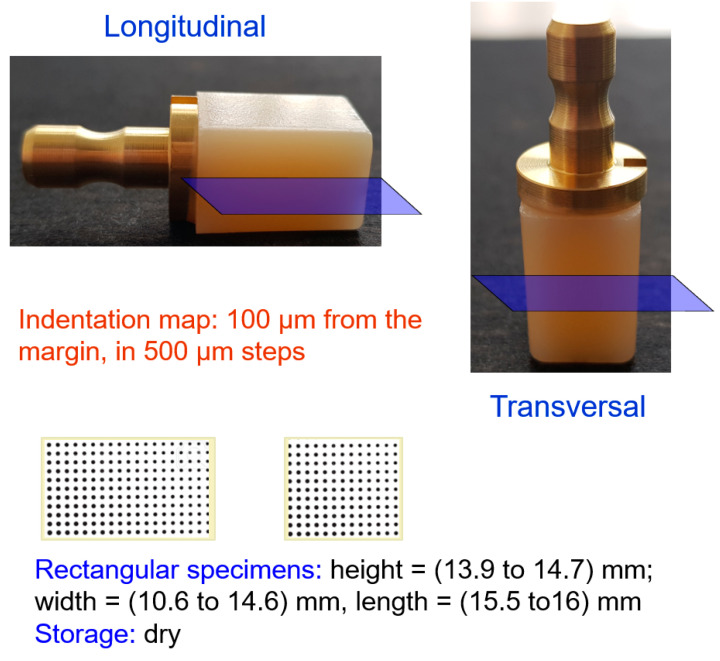
Section (longitudinal and transversal) and dimension of the CAD/CAM RBCs area deployed for depth-sensing indentation test.

Longitudinal and transversal sections were cut (Isomet low-speed saw, Buehler, Germany) with water cooling through the center of CAD/CAM blocks (Figure 1). Each sample was then fixed to a glass slide and mounted in an automatic grinding machine (EXAKT 400CS Micro Grinding System EXAKT Technologies Inc. Oklahoma, OK, USA). Specimens were wet-ground with silicon carbide sand paper (grit size p1200, p2500, and p4000, LECO Corporation, St. Joseph, MI, USA) and polished with a diamond suspension (mean grain size: 1 µm) for 2–3 min, until each surface was shiny.

The dimensions of the sample corresponded to the type of section (longitudinal and transversal) and the dimensions of the individual CAD/CAM blocks, which varied between the individual brands in the range 13.9 to 14.7 mm, 10.6 to 14.6 mm, and 15.5 to 16 mm, respectively.

To enable a comparison with the tooth structure, five caries-free human molars were selected. Anonymized teeth have been used for this purpose. Each tooth was cut in the middle and perpendicular to the longitudinal axis of the tooth. A second parallel cut was made, providing a 2 mm plane-parallel sample for each tooth. The samples were fixed on a glass slide, the first section was exposed and then ground and polished as described above.

In addition, filler size and shape were analyzed by scanning electron microscopy.

### 2.2. Depth-Sensing Indentation Parameters

A series of micro-mechanical parameters were calculated from depth-sensing indentations (automatic micro-hardness indenter, Fischerscope H100C, Fischer, Germany) according to DIN 50359-1:1997-10 [20]. Measurements were made in CAD/CAM sections in 500 µm steps to create an area mapping comprising 600 to 900 indentations per each specimen, given by the individual specimen geometry. In tooth specimens, 20 randomly chosen indentations per specimen were performed in both enamel and dentin.

The indentation was carried out force controlled; the test load increased within 20 s and decreased within 20 s, with constant speed between 0.4 mN and 500 mN. The load and the penetration depth of the indenter were continuously measured during the load–unload hysteresis. The force resolution of the device is lower than 150 nN and the path resolution lower than 10 pm. During the measurement, the indenter is driven into the material, and both elastic and plastic deformation processes occur. This produces an impression with a projected area (A_p_) and a surface area (A_s_). The universal hardness (Martens hardness, HM = F/A_s_(h)) is defined as the test force divided by the surface area, A_s_, of the indentation under the applied test force. The values indicated in the results correspond to the maximal load (F_max_ = 500 mN). This hardness definition includes both plastic and elastic deformation of the material tested. For indentation depth < 6 µm, which relates to the measurements of the present study, the surface area A_s_(h) cannot be assumed to be that of an ideal shaped Vickers indenter because of the rounding at the tip. Therefore, the exact area function (mathematical function relating the surface area A_s_(h) to the distance from the indenter tip) for the used indenter was determined according to Oliver and Pharr [21] and ISO/DIS 14577 [22]. The indentation hardness (H_IT_ = F_max_/A_p_) is a measure of the resistance to plastic deformation and was used to calculate the Vickers hardness (HV). The relation between indentation hardness H_IT_ and Vickers Hardness is defined as: HV = 0.0945 x H_IT_ and was implemented in the software, so that the measurement results were indicated in the more familiar Vickers hardness (HV) units. The indentation modulus (Y) was calculated from the slope of the tangent of indentation depth-curve at maximum force. The mechanical work W_total_ indicated during the indentation procedure is only partly consumed as plastic deformation work W_plast_. During the removal of the test force the remaining part is set free as work of the elastic reverse deformation W_elastic_. According to the definition of the mechanical work as W = ∫F dh (F = load; h = indentation depth) and considering the force variation during load and unload, the total mechanical work and its components were calculated.

By measuring the change in indentation depth with constant test force, a relative change of the indentation depth could also be calculated. This value describes the creep, Cr, of the material as (h_2_−h_1_)/h_1_ [20]_,_ with h_1_ = indentation depth when the test force reached 500 mN, and h_2_ = indentation depth measured after 5 s of holding a constant force of 500 mN.

**Figure 2:**
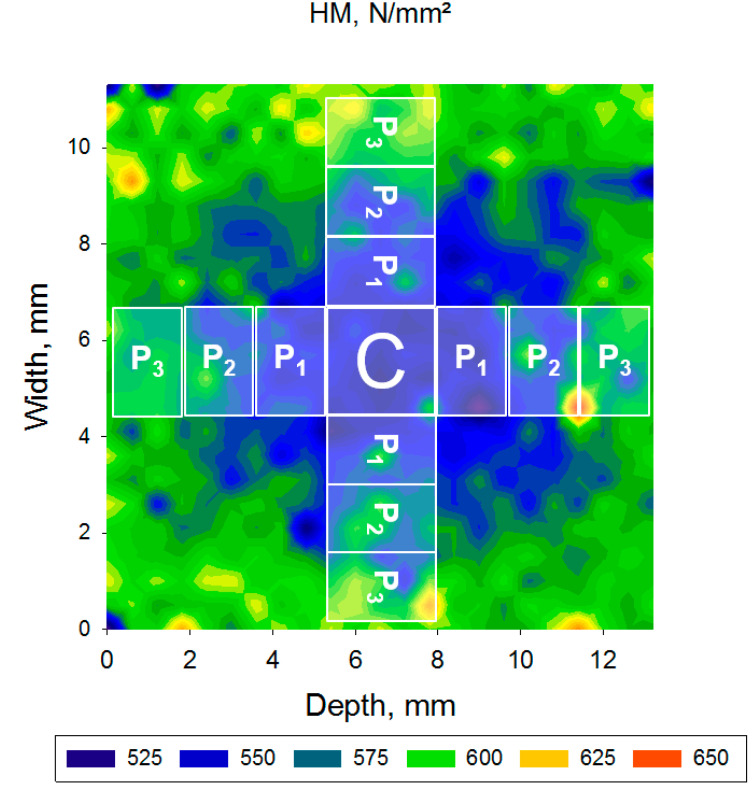
Allocation of the central (C) and peripheral (P) zones in a data map using the example of the variation of the parameter Martens hardness (HM) in the material SB.

For statistical purposes, a central zone (C) of 3 × 3 mm^2^ was defined as a reference in each mapping area (Figure 2). Starting from this central zone, peripheral zones (P_x_, with x = 1 to 4) were defined in steps of 1.5 mm wide and 3 mm long. The number of peripheral zones depends on the dimensions of the CAD/CAM blocks.

### 2.3. Scanning Electron Microscopy (SEM)

One specimen of each material was selected for the SEM analysis (Zeiss Supra 55VP. Oberkochen, Germany). Specimens were unsputtered, while the images were taken using a backscatter signal (RBSD).

### 2.4. Statistical Analyses

The distribution of the variables was tested with the Shapiro–Wilk procedure. All variables were normally distributed, and a parametric approach was used. The results were compared using one- and multiple-way analysis of variance (ANOVA) and Tukey’s honestly significant difference (HSD) post hoc-test (α = 0.05) using an alpha risk set at 5% (SPSS Inc. Version 25.0, Chicago, IL, USA). A multivariate analysis (general linear model) assessed the effect of various parameters as well as their interaction terms on the analyzed micro-mechanical properties. The partial eta-squared statistic reported the practical significance of each term, based on the ratio of the variation attributed to the effect. Larger values of partial eta-squared (η_P_²) indicate a greater amount of variation accounted for by the model, which add up to a maximum of 1.0.

## 3. Results

The cumulative results of the depth-sensing indentation within a material enable the identification of significant differences in the micro-mechanical properties among the tested materials (Table 2). A significant direct correlation is observed among filler amount and the depth-sensing parameters HM (Pearson correlation coefficient, *p* = 0.931), HV (*p* = 0.912), and Y (*p* = 0.923), and there is a significant inverse correlation among filler amount and depth-sensing parameters Cr (*p* = −0.708), W_e_ (*p* = −0.925) and W_tot_ (*p* = −0.950). Correspondingly, the significant highest HM, HV and Y (*p* < 0.001), and lowest Cr, W_e_ and W_tot_ (*p* < 0.001) were observed in the highest filled CAD/CAM RBC GB, while the opposite is valid for SB, which is the material with the lowest filler amount. In line with these results, the multivariate analysis (general linear model) identified a very strong influence of the CAD/CAM RBC on all measured properties (*p* < 0.001; partial eta squared η_P_^2^ > 0.943), while the most sensitive parameters in identifying differences among materials were Y (*p* < 0.001; η_P_^2^ = 0.987) and W_e_ (*p* < 0.001; η_P_^2^ = 0.987).

**Figure 3:**
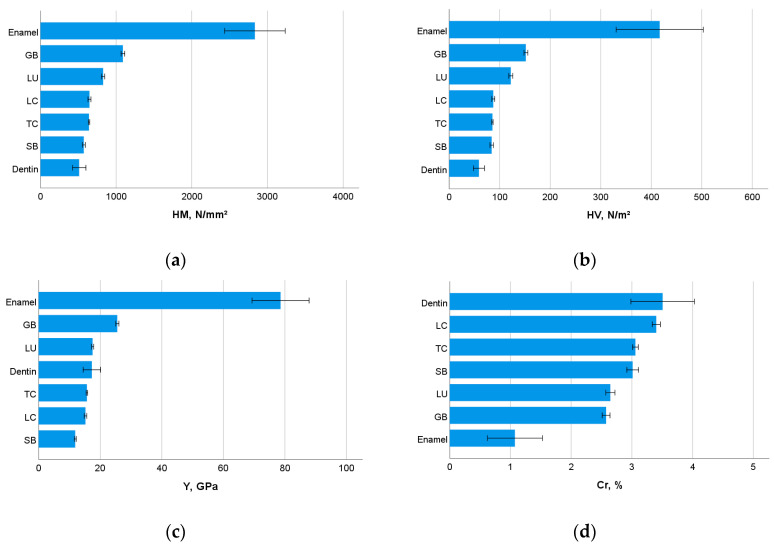
Comparative representation of the depth-sensing indentation parameters ((**a**) Martens Hardness, HM; (**b**) Vickers hardness, HV; (**c**) indentation modulus, Y; (**d**) creep, Cr) of the tooth structure (enamel and dentin) and CAD/CAM RBCs.

The analyzed CAD/CAM RBCs are intended to replace the tooth structure. Figure 3 therefore illustrates, comparatively, the depth-sensing parameters HM, HV, Y, and Cr, measured in enamel, dentin, and the analyzed CAD/CAM RBCs. One-way ANOVA identified in enamel the significantly highest HM, HV and Y values (*p* < 0.001), and the significantly lowest Cr values (*p* < 0.001). In contrast, dentin shows the lowest HM and HV values (*p* < 0.001) and the significantly highest Cr (*p* < 0.001). It ranks statistically with respect to Y in the middle of the CAD/CAM RBCs sequence presented in Table 2, while showing statistically similar values to LU (*p* = 0.110).

**Figure 4:**
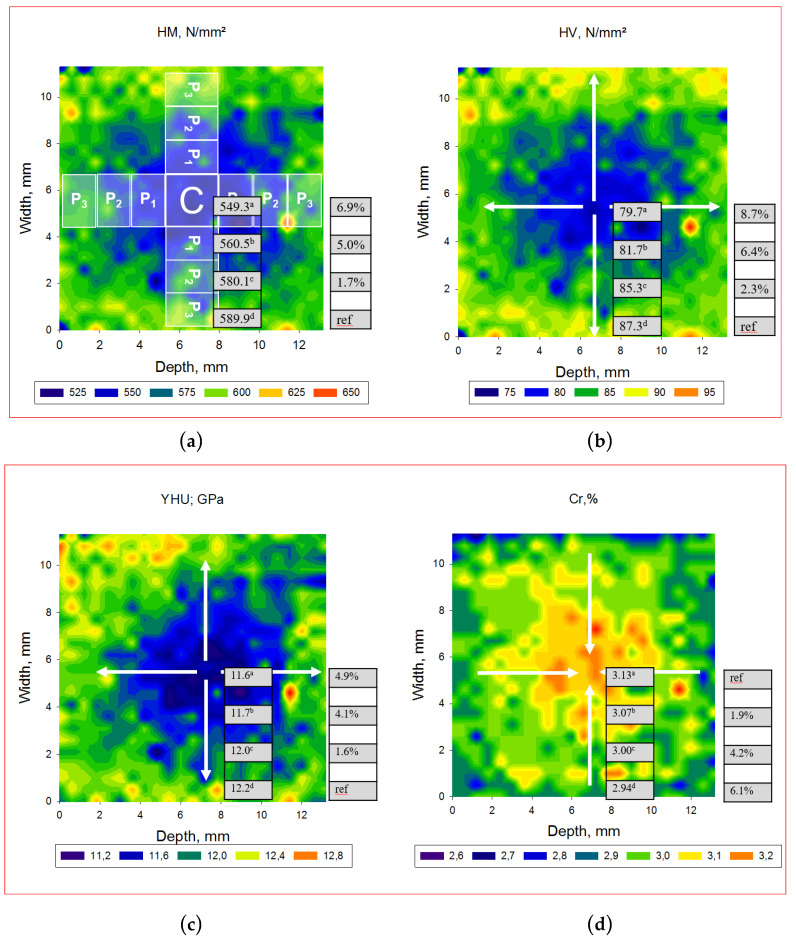
Spatial distribution of the depth-sensing indentation parameters ((**a**) Martens Hardness, HM; (**b**) Vickers hardness, HV; (**c**) indentation modulus, Y; (**d**) creep, Cr) exemplified on a transversal section performed in an SB CAD/CAM block. Mean values of the parameters corresponding to the central (C) and peripheral zones (P_1_, P_2_, and P_3_) are specified, while superscripts indicate statistically homogeneous subgroups (Tukey’s HSD test, α = 0.05). The percentage decrease in HM, HV, and Y towards the central zone is related to the outer peripheral zone (P_3_). The central zone (C) was the reference used to calculate the percentage decrease towards the periphery for Cr.

**Figure 5:**
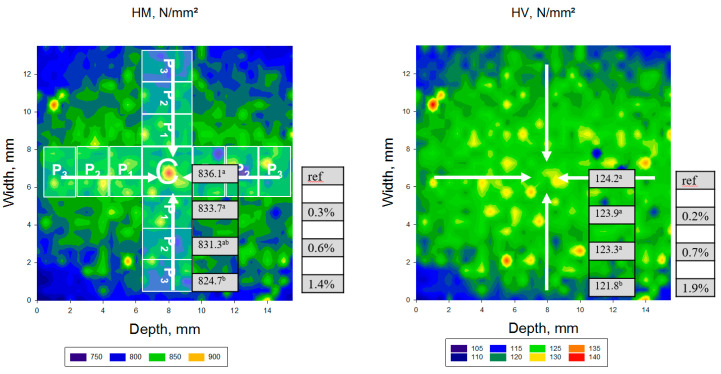
Spatial distribution of the depth-sensing indentation parameters (Martens Hardness, HM; Vickers hardness, HV) exemplified on a transversal section performed in an LU CAD/CAM block. Mean values of the parameters corresponding to the central (C) and peripheral zones (P_1_, P_2_, and P_3_) are specified, while superscripts indicate statistically homogeneous subgroups (Tukey’s HSD test, α = 0.05). The percentage decrease in HM, HV, and Y towards the peripheral zone is related to the central zone (C). The reference to calculate the percentage increase towards the central zone for Cr was the outer peripheral zone (P_3_).

**Figure 6:**
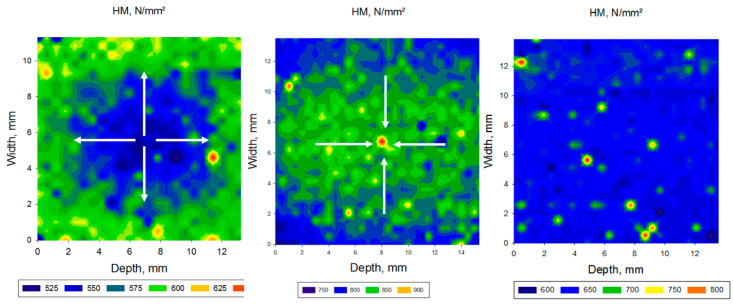
Comparative spatial distribution pattern of the Martens Hardness (HM) exemplified on SB (left), LU (middle), and TC (right).

The spatial distribution of the depth-sensing indentation parameters within transversal and longitudinal sections performed in the CAD/CAM RBC block is illustrated in Figure 4, Figure 5 and Figure 6 and Table 3, Table 4, Table 5, Table 6 and Table 7. Parameters measured in transversal and longitudinal sections were statistically similar within one material and zone. One-way ANOVA identified three different patterns of variation of the measured parameters in the analyzed CAD/CAM RBC blocks, based on the location of measurement. Significantly higher HV, HM, Y and corresponding lower Cr, W_e_ and W_tot_ were identified in the central compared to the peripheral region in LU, GB, and TC. The opposite is valid for SB, whereas the values measured in LC in central and peripheral zones were mostly statistically similar. Details of these variations are provided below for each CAD/CAM RBC, in the ascending order of the filler amount.

In SB blocks (Figure 4 and Table 3), the significantly highest HV, HM and Y values were identified at the periphery of the CAD/CAM block, while they decreased significantly towards the center. In relation to the outer peripheral zone, this decrease is 4.9% for Y, 6.9% for HM and 8.7% for HV. The parameters Cr, W_e_ and W_tot_ varied in reverse order and reached the highest values in the central area.

The variation pattern in the measured depth-sensing indentation parameters is different in TC (Table 4). The significantly highest HV, HM, and Y values were identified in the center of the CAD/CAM block, while they decreased significantly towards the periphery. A corresponding inverse relationship was observed for the parameters Cr, W_e_ and W_tot._ Note that the differences among the central and peripheral zones are very small compared to the material previously presented (SB) and the delimitation of the zones is less accentuated. The central and the four peripheral zones are statistically divided into only two homogeneous subgroups, while the material (SB) previously presented contained four subgroups.

The trend in the variation pattern of measured properties in LC (Table 5) is similar to that of TC, but the variation between zones is even less, while for some parameters it is even outside the significance limit.

LU (Figure 5, Table 6) also followed a similar pattern in varying the measured properties within the zones, with the central and peripheral zone differences being less than 2% in the transversal section. Note that the outer peripheral zone P4 in the longitudinal section, which is larger than the transverse zone, showed a significantly higher variation in the measured properties compared to the central measurement location. However, the percentage decrease in HM, HV and Y in the direction of the peripheral zone did not exceed 3.3%, 4% and 1.7%, respectively, in longitudinal section.

A slight inhomogeneity among the measured parameters is also observed in GB (Table 7), whereby slightly, but significantly, higher HM, HV and Y values are identified at the central location compared to the periphery, while the parameters Cr, W_e_ and W_tot_ remained unchanged.

Figure 6 is illustrating comparatively the three-variation pattern in the analyzed CAD/CAM blocks exemplified for the parameter HM, showing a low (up to 8.7%) increase in properties towards periphery (SB), a low (up to 4%) decrease in properties towards periphery (LU, TC, GB), and constant, statistical similar properties in LC.

**Figure 7:**
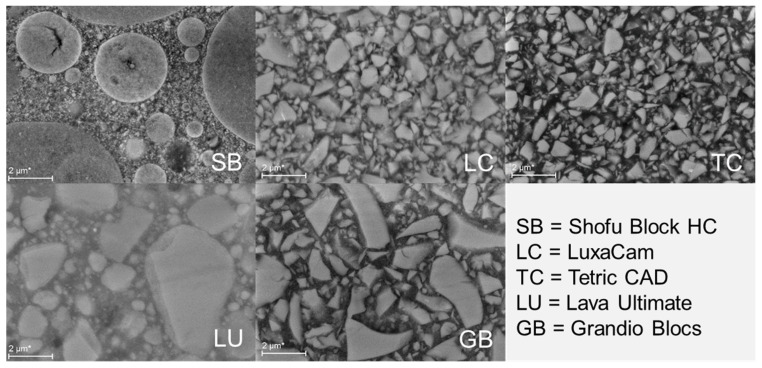
Scanning electron microscopy (SEM) on unsputtered CAD/CAM RBC specimens.

Figure 7 summarizes the scanning electron microscopic images of the analyzed CAD/CAM RBCs. In addition to the large differences in filler loading and chemical composition, which are indicated in Table 1, the filler morphology of the analyzed materials also differs. Spherical particles, with a diameter of several µm, and a multimodal particle size distribution are thus identified in SB. They show a densified cluster appearance, which is similar to the zirconia/silica cluster observed in LU but differs in shape. In addition to the zirconia/silica micro-clusters, the filling system in LU also contains silica and zirconia nano-fillers. The backscattering method used for the SEM analysis enable a differentiation between the various dense Zr (white) and Si (gray) oxide nano-fillers that were compacted into micro-clusters. In contrast, LC, TC and GB contain irregular glass fillers that are larger in GB and very similar in size and distribution in LC and TC. For TC, the manufacturer specifies them as BaO-Al_2_O_3_-SiO_2_-glass fillers, while for LC and GB the specifications given by the manufacturer are not exact.

## 4. Discussion

The data allow both null hypotheses to be rejected. CAD/CAM RBC blocks showed significantly different depth-sensing parameters, while the spatial distribution of properties within a block indicates gradually varying properties that can increase or decrease from central regions to the periphery. The degree of variation in the measured depth-sensing parameters is material-specific and less than 8.7%.

The analyzed materials are heterogeneous composite materials based on a highly cross-linked poly(dimethacrylate) network with embedded inorganic particles of irregular shape and uneven distribution (Figure 7). Poly(dimethacrylate) networks are also described as spatially heterogeneous when related to their microstructure, which consists of highly cross-linked microgel agglomerates and less cross-linked areas [23]. In most materials analyzed, the polymer matrix is the result of the copolymerization of different types and ratios of monomer molecules (Table 1). In addition to varying chain length and stereochemistry, copolymers differ in their composition (the relative amounts of each monomer incorporated into the copolymer), sequence distribution (the way in which these monomers are arranged within the chain), and architecture (linear, graft, branched). The heterogeneity in dimethacrylate networks has also been associated with hydrogen bonding in the polymerization system and is therefore considered the lowest in TEGDMA networks that do not have groups that can form strong hydrogen bonds [24]. Literature reports suggest that the degree of heterogeneity influences the mechanical properties of the polymers and the high brittleness of poly(dimethacrylates) can be attributed to their heterogeneous nature [25].

The spatial distribution of the micro-mechanical properties was carried out using a depth-sensing indentation test. In this type of test, the applied load and the depth of penetration of the Vickers indenter into the sample were recorded in real time and used to indirectly determine the contact area and thus the hardness of the sample. The contact equations also enable the indentation modulus of the materials and various elastic/plastic parameters to be determined, as described above [20,21]. The best performance from a mechanical point of view is that the highest HV, HM, Y and the lowest Cr and plastic deformation are reached in one material. Indentation tests were performed at relatively high load/depth in order to avoid intrinsic micro-structural heterogeneity. The depth of indentation at the maximum load of 500 mN recorded over all measured materials and zones was (5.27 ± 0.61) µm. With Vickers indentation, the indentation size (indentation diagonals) can be rated as approximately seven times greater than the indentation depth measured at maximum load. In the analyzed CAD/CAM RBCs, this value ranges from approximately 33 µm to 42 µm and is therefore a multiple of the largest filler size identified in the SEM analysis (Figure 7). In view of this, the depth-sensing test averages the local inhomogeneity between filler and organic matrix and thus enables the effective mechanical properties of the material and not the mechanical properties of its individual components (filler, matrix) to be assessed. This endorsed identifying variations of the average values within different locations in a CAD/CAM block. Moreover, according to Hertz’s contact theory, the mechanical properties measured by indentation involve a semi-ellipsoidal volume extending to about nine times the indentation depth in vertical direction and about seven times the depth in radial direction [26]. For an indentation depth of ~5 µm, this represents a material volume of 45 µm in depth and 35 µm in the lateral direction. Thus, indent positions were chosen to match the lateral deformation and were placed at a distance of 500 µm in both x and y direction, while the specimen thickness amounted to half of the entire CAD/CAM block (>7 mm).

In the analyzed CAD/CAM RBCs, the hardness parameters HM and HV increased progressively with increasing amount of inorganic filler. The indentation modulus Y follows this sequence with a slight deviation, which was found for the materials TC and LC (Table 2). Both materials have a very similar filler content (71.1% by weight compared to 70% by weight, Table 1), size and morphology (Figure 7). The small deviation in the sequence of the materials by increasing the amount of filler in Y compared to HM and HV most probably occurred because the filler weight was taken into account instead of the filler volume. The latter has a stronger influence on the modulus of elasticity in RBCs [27], but the information about the filler volume is not available. The results are in line with the optical properties previously determined for the same materials and shades, that follow the material sequence observed for the indentation modulus [15]. Accordingly, a higher linear absorption coefficient was identified in LC compared to TC [15] that accounts for a higher light scattering in the material with the lower filler (% by weight) amount (LC). The linear absorption coefficient was determined by measuring the materials’ absorbance using a dental light curing unit for exposure [15]. Scattering of light occurs in RBCs when the light is deviated from its initial trajectory by localized non-uniformities with a different index of refraction, e.g., reinforcing particles, or porosity voids [28]. It should be noted that the light scatter also increases with the mismatch of the refractive index between the fillers and the methacrylate matrix [29,30]. By this observation, it can be inferred that the higher light scattering observed in LC (70 wt.%) vs. TC (71.1 wt.%) can be related to small differences in the chemical composition of the individual RBC compounds. This can either have led to a higher filler volume (lower density) or/and to differences in the refractive index of the individual components and/or to their mismatch. The relationship can only be speculated due to the imprecise specification of the chemical composition of the analyzed materials. For LC in particular, the filling system is only vaguely referred to as containing SiO_2_-glasses, and the organic matrix is generically specified as methacrylate based.

The integral of the force–displacement curves during the indentation process shows that the mechanical work W_tot_ in the analyzed materials progressively decreases with increasing amount of inorganic filler and is therefore the lowest in GB and the highest in SB. It follows thus, similar to Cr, an inverse variation pattern in comparison to the parameters HM, HV and E discussed above. This mechanical work is only partially consumed as plastic deformation work, while the remaining part is released as work of the elastic reverse deformation, W_e_. Notably, the individual parts of W_tot_, namely the plastic deformation work and the elastic deformation work, correlate somewhat less than W_tot_ with the inorganic filler weight, which, similar to the discussion above on Y, indicates an influence of the filler volume amount and the filler size.

CAD/CAM RBCs restorations are intended to replace the tooth structure. It is therefore surprising that their properties differ to such a high extent. The present study identified HM, HV, and Y values that are more than twice as high, or more than twice as low (Cr) in the tooth enamel than the analyzed materials and dentine. This result is linked to the chemical composition and the microstructure of enamel, which is a highly mineralized and crystalline material that contains a significantly higher amount of inorganic fillers (hydroxyapatite, 98% by weight) [31] than any of the materials analyzed (Table 1). In contrast, dentin can be seen as a complex nanocomposite, in which nanoparticles made of hydroxyapatite are embedded in organic collagen fibrils that are burned to different degrees, while the inorganic amount (70–72 wt.%, 40–45 vol.%) [31] is much closer to the filler weight amount in the materials analyzed (Table 1). The indentation modulus recorded for the tooth structure validates previously published data [32]. It may be interesting to note that the dentin behaves differently in terms of hardness and indentation modulus. It fits well into the CAD/CAM RBC sequence, based on the amount of inorganic filler for the indentation modulus, but offers the significantly lowest hardness.

The spatial distribution of the depth-sensing indentation parameters within the CAD/CAM RBC block identified two opposite patterns of variation in the measured properties, while in one material the properties were almost similar. There is clear evidence of a gradual variation in properties from the center of the block to peripheral locations in almost all RBC blocks, which may be related to the order in which the blocks are exposed to pressure and/or curing light [17]. The limited literature on the manufacture of RBC CAD/CAM blocks for dental restoration indicates that many systems currently on the market use for initiating the polymerization both light and heat catalysts in the same resin [17]. The gradual decrease (HV, HM, Y) and the corresponding increase (Cr, W_e_ and W_tot_) in properties towards the center observed in SB may be related to light attenuation during polymerization, temperature and pressure gradients, and most likely to accumulated residual stress. The latter is inevitably introduced into the surface layer during forming and heat treatment while a compressive residual stress into the surface layer may improve the mechanical properties [33]. A number of other parameters need to be considered in relation to residual stress accumulation, such as the matrix shrinkage during polymerization, the different rate of shrinkage across the block cross-section, the gelation point, the degree of cross-linking, thermal shrinkage, or the cool-down rate. It should be emphasized that SB is the material with the highest monomer content, which may be the reason why the observed effects are more prominent in this material.

The partially lower HV, HM, Y and correspondingly higher Cr, W_e_ and W_tot_ identified in LU, GB, TC and LC near the outer surface of the CAD/CAM block compared to the center of the block can partly be due to the high sensitivity of the radical polymerization toward oxygen inhibition, especially if the polymerization was carried out in air-saturated media or in an air atmosphere or when oxygen sensitizers or oxygen scavenging additives were not taken into account [34].

The different behavior in the variation of the spatial-distribution of the micro-mechanical properties in CAD/CAM blocks cannot be related to either the amount of filler or the chemical composition of the fillers and the organic matrix, and most likely depends on the manufacturing process and the strategies involved in conducting the polymerization process. Since five different materials were randomly selected and tested in the present study, differences between brands from different manufacturers must be assumed.

## 5. Conclusions

CAD/CAM RBC blocks show gradually varying properties that can increase or decrease from the central regions to the periphery without any direct reference to the chemical composition of the materials or the inorganic filler fraction. The degree of variation in the measured micro-mechanical parameters is material-specific and less than 8.7%. Both clinical applications and in vitro study designs should consider that there is a slight inhomogeneity of the micro-mechanical properties in CAD/CAM RBC blocks.

## Figures and Tables

**Table 1:** Analyzed computer-aided design/computer-aided manufacturing (CAD/CAM) resin-based composites (RBCs): abbreviation, name, manufacturer, shade, LOT and composition, as indicated by the manufacturer.

Code	CAD/CAM RBC	Manufacturer	Shade	LOT	Monomer	Filler	
						Composition	wt.%
SB	Shofu Block HC	Shofu	A3 HT	071601	UDMA TEGDMA	SiO_2_, silicate, zirconium silicate	61
LC	Luxacam Composite	DMG	A3	769515	Methacrylates	SiO_2_-glass	70
TC	Tetric CAD	Ivoclar Vivadent	A3 HT	W93631	Bis-GMA UDMA Bis-EMA TEGDMA	BaO-Al_2_O_3_-SiO_2_-glass, SiO_2_	71.1
LU	Lava Ultimate	3M	A3 HT	N933699	Bis-GMA UDMA Bis-EMA TEGDMA	SiO_2_, ZrO_2_, aggregated ZrO_2_/SiO_2_ cluster	80
GB	Grandio Blocs	Voco	A3 HT	1709591	Methacrylates	n.a.	86

Abbreviations: Bis-GMA = bisphenol A glycol dimethacrylate; Bis-EMA = ethoxylated bisphenol A dimethacrylate; TEGDMA = Triethylene glycol dimethacrylate; UDMA = Urethane dimethacrylate; SiO_2_ = silicon oxide (silica); ZrO_2_ = zirconium oxide.

**Table 2:** Variation of the micro-mechanical properties (Martens Hardness, HM; Vickers hardness, HV; indentation modulus, Y; creep, Cr; elastic indentation work, W_e_; total indentation work, W_tot_) among tested materials (mean value and standard deviation below the mean value). Output data include all individual indentations and are arranged in ascending order of the values HM. Superscripts indicate statistically homogeneous subgroups within one column; Tukey’s HSD test, α = 0.05. The filler amount in weight % is also indicated.

CAD/CAM RBC	HM [N/m²]	HV [N/m²]	Y [GPa]	Cr [%]	W_e_ [µJ]	W_tot_ [µJ]	Filler [wt.%]
SB	573.2 ^A^	84.2 ^A^	11.9 ^A^	3.01 ^C^	0.57 ^E^	0.99 ^E^	61
	19.1	3.4	0.3	0.10	0.01	0.02	
TC	641.3 ^B^	85.7 ^B^	15.7 ^C^	3.06 ^D^	0.45 ^B^	0.93 ^D^	71.1
	9.2	1.3	0.2	0.05	0.00	0.00	
LC	647.7 ^C^	87.3 ^C^	15.2 ^B^	3.40 ^E^	0.47 ^D^	0.93 ^C^	70
	19.3	2.9	0.4	0.07	0.01	0.01	
LU	828.0 ^D^	122.0 ^D^	17.5 ^D^	2.65 ^B^	0.45 ^C^	0.82 ^B^	80
	20.2	3.9	0.3	0.07	0.00	0.01	
GB	1089.4 ^E^	151.9 ^E^	25.6 ^E^	2.58 ^A^	0.35 ^A^	0.72 ^A^	86
	23.7	3.8	0.5	0.06	0.00	0.01	

**Table 3:** SB: variation of the micro-mechanical properties (Martens Hardness, HM; Vickers hardness, HV; indentation modulus, Y; creep, Cr; elastic indentation work, W_e_; total indentation work, W_tot_) among the analyzed central (C) and peripheral (P) zones in a transversal (T) and longitudinal (L) section in SB CAD/CAM blocks (mean values and standard deviation below). Superscripts/subscripts indicate statistically homogeneous subgroups within one column; Tukey’s HSD test, α = 0.05.

SB	HM		HV		Y		Cr		W_e_		W_tot_	
	T	L	T	L	T	L	T	L	T	L	T	L
C	549.3 ^a^	549.4 _a_	79.7 ^a^	79.5 _a_	11.6 ^a^	11.6 _a_	3.13 ^a^	3.17	0.57 ^a^	0.57 _a_	1.01 ^a^	1.01 _a_
	12.4	9.7	2.1	1.6	0.2	0.2	0.06	0.05	0.01	0.01	0.01	0.01
P1	560.5 ^b^	552.5 _a_	81.7 ^b^	80.2 _a_	11.7 ^b^	11.6 _a_	3.07 ^b^	3.15	0.57 ^ab^	0.57 _a_	1.00 ^b^	1.01 _a_
	14.8	11.7	2.5	2.1	0.2	0.2	0.06	0.06	0.01	0.01	0.01	0.01
P2	580.1 ^c^	568.6 _b_	85.3 ^c^	83.5 _b_	12.0 ^c^	11.8 _b_	3.00 ^c^	3.03	0.56 ^b^	0.57 _b_	0.98 ^c^	0.99 _a_
	15.7	17.0	2.8	3.0	0.2	0.3	0.07	0.08	0.01	0.01	0.01	0.01
P3	589.9 ^d^	573.0 _bc_	87.3 ^d^	84.4 _bc_	12.2 ^d^	11.9 _b_	2.94 ^d^	2.99	0.56 ^b^	0.57 _c_	0.97 ^d^	0.99 _a_
	16.1	15.4	3.1	2.7	0.2	0.3	0.08	0.09	0.01	0.01	0.02	0.01
P4		577.2 _c_		85.2 _c_		11.9 _b_		2.97		0.57 _c_		0.98 _a_
		19.3		3.1		0.3		0.08		0.01		0.02

**Table 4:** TC: variation of the micro-mechanical properties (Martens Hardness, HM; Vickers hardness, HV; indentation modulus, Y; creep, Cr; elastic indentation work, W_e_; total indentation work, W_tot_) among the analyzed central (C) and peripheral (P) zones in a transversal (T) and longitudinal (L) section in TC CAD/CAM blocks. Superscripts/subscripts indicate statistically homogeneous subgroups within one column; Tukey’s HSD test, α = 0.05.

TC	HM		HV		Y		Cr		W_e_		W_tot_	
	T	L	T	L	T	L	T	L	T	L	T	L
C	646.6 ^a^	643.5 _a_	86.5 ^a^	85.8 _a_	15.8 ^a^	15.8 _a_	3.06 ^a^	3.06 _a_	0.45 ^a^	0.45 _a_	0.93 ^a^	0.93 _a_
	6.3	5.8	0.9	0.7	0.1	0.2	0.02	0.03	0.00	0.00	0.00	0.00
P1	646.8 ^a^	638.6 _b_	86.6 ^a^	85.4 _a_	15.8 ^ab^	15.6 _a_	3.06 ^ab^	3.06 _ab_	0.45 ^a^	0.45 _a_	0.93 ^ab^	0.93 _a_
	8.0	5.0	1.3	0.6	0.1	0.2	0.04	0.03	0.00	0.00	0.00	0.00
P2	643.0 ^ab^	640.3 _b_	85.9 ^ab^	85.5 _a_	15.7 ^ab^	15.7 _a_	3.07 ^ab^	3.07 _ab_	0.45 ^a^	0.45 _a_	0.93 ^ab^	0.93 _a_
	15.1	6.0	2.2	0.8	0.3	0.2	0.06	0.04	0.00	0.01	0.01	0.00
P3	639.5 ^b^	641.0 _b_	85.4 ^b^	85.5 _a_	15.7 ^b^	15.7 _a_	3.08 ^b^	3.07 _ab_	0.45 ^a^	0.45 _a_	0.93 ^b^	0.93 _a_
	10.0	7.1	1.4	1.0	0.3	0.2	0.04	0.05	0.01	0.00	0.01	0.00
P4		639.6 _b_		85.6 _a_		15.6 _a_		3.08 _b_		0.45 _a_		0.93 _a_
		6.5		1.0		0.2		0.05		0.01		0.00

**Table 5:** LC: variation of the micro-mechanical properties (Martens Hardness, HM; Vickers hardness, HV; indentation modulus, Y; creep, Cr; elastic indentation work, W_e_; total indentation work, W_tot_) among the analyzed central (C) and peripheral (P) zones in a transversal (T) and longitudinal (L) section in LC CAD/CAM blocks. Superscripts/subscripts indicate statistically homogeneous subgroups within one column; Tukey’s HSD test, α = 0.05.

LC	HM		HV		Y		Cr		W_e_		W_tot_	
	T	L	T	L	T	L	T	L	T	L	T	L
C	643.4 ^a^	658.4 _a_	86.8 ^a^	88.7 _a_	15.1 ^a^	15.5 _a_	3.40 ^a^	3.38 _a_	0.47 ^a^	0.46 _a_	0.93 ^a^	0.92 _a_
	8.8	8.1	1.3	1.2	0.2	0.3	0.04	0.04	0.00	0.00	0.01	0.01
P1	644.6 ^a^	658.3 _a_	87.1 ^a^	88.8 _a_	15.1 ^a^	15.5 _a_	3.40 ^ab^	3.38 _ab_	0.47 ^a^	0.46 _a_	0.93 ^a^	0.92 _a_
	9.4	12.6	1.4	2.0	0.2	0.3	0.04	0.04	0.00	0.00	0.01	0.01
P2	644.2 ^a^	654.3 _ab_	87.0 ^a^	88.2 _ab_	15.1 ^a^	15.4 _ab_	3.41 ^ab^	3.39 _ab_	0.47 ^a^	0.46 _a_	0.93 ^a^	0.92 _a_
	13.5	16.9	2.0	2.6	0.3	0.4	0.05	0.06	0.01	0.01	0.01	0.01
P3	646.8 ^a^	648.2 _bc_	87.2 ^a^	87.2 _bc_	15.1 ^a^	15.3 _bc_	3.40 ^ab^	3.40 _ab_	0.47 ^a^	0.46 _a_	0.93 ^a^	0.92 _a_
	16.7	15.7	2.5	2.3	0.4	0.4	0.06	0.06	0.01	0.01	0.01	0.01
P4	642.4 ^a^	643.8 _c_	86.8 ^a^	86.7 _c_	15.0 ^a^	15.1 _c_	3.43 ^b^	3.41 _b_	0.47 ^a^	0.47 _b_	0.93 ^a^	0.93 _b_
	18.8	29.1	2.8	4.3	0.4	0.5	0.07	0.11	0.01	0.01	0.01	0.01

**Table 6:** LU: variation of the micro-mechanical properties (Martens Hardness, HM; Vickers hardness, HV; indentation modulus, Y; creep, Cr; elastic indentation work, W_e_; total indentation work, W_tot_) among the analyzed central (C) and peripheral (P) zones in a transversal (T) and longitudinal (L) section in LU CAD/CAM blocks. Superscripts/subscripts indicate statistically homogeneous subgroups within one column; Tukey’s HSD test, α = 0.05.

LU	HM		HV		Y		Cr		W_e_		W_tot_	
	T	L	T	L	T	L	T	L	T	L	T	L
C	836.1 ^a^	843.1 ^a^	124.2 ^a^	124.5 _a_	17.5 ^a^	17.9 _a_	2.6 ^a^	2.59 _a_	0.45 ^a^	0.45 _a_	0.82 ^a^	0.82 _a_
	16.7	21.6	3.5	4.3	0.2	0.3	0.05	0.05	0.00	0.00	0.01	0.01
P1	833.7 ^a^	839.1 _ab_	123.9 ^a^	123.8 _ab_	17.5 ^a^	17.8 _a_	2.61 ^a^	2.62 _a_	0.45 ^ab^	0.45 _a_	0.82 ^a^	0.82 _ab_
	13.0	9.7	2.6	1.9	0.2	0.1	0.05	0.06	0.00	0.00	0.01	0.01
P2	831.3 ^ab^	833.6 _bc_	123.3 ^a^	122.7 _bc_	17.5 ^a^	17.7 _ab_	2.64 ^a^	2.62 _ab_	0.45 ^ab^	0.45 _a_	0.82 ^ab^	0.82 _ab_
	15.8	15.5	3.3	2.8	0.2	0.3	0.06	0.07	0.001	0.001	0.01	0.01
P3	824.7 ^b^	826.8 _c_	121.8 ^b^	121.5 _c_	17.4 ^a^	17.6 _b_	2.67 ^b^	2.65 _b_	0.45 ^b^	0.45 _a_	0.83 ^b^	0.82 _b_
	21.2	24.4	4.0	4.9	0.3	0.3	0.08	0.07	0.00	0.00	0.01	0.01
P4	-	815.0 _d_	-	119.2 _d_	-	17.4 _c_	-	2.69 _c_	-	0.45 _a_	-	0.83 _c_
		19.9		3.5		0.4		0.08		0.001		0.01

**Table 7:** GB: variation of the micro-mechanical properties (Martens Hardness, HM; Vickers hardness, HV; indentation modulus, Y; creep, Cr; elastic indentation work, W_e_; total indentation work, W_tot_) among the analyzed central (C) and peripheral (P) zones in a transversal (T) and longitudinal (L) section in GB CAD/CAM blocks. Superscripts/subscripts indicate statistically homogeneous subgroups within one column; Tukey’s HSD test, α = 0.05.

GB	HM		HV		Y		Cr		W_e_		W_tot_	
	T	L	T	L	T	L	T	L	T	L	T	L
C	1108.0 ^a^	1088.4 _a_	154.9 ^a^	151.6 _a_	25.9 ^a^	25.6 _a_	2.54 ^a^	2.61 _a_	0.35 ^a^	0.35 _a_	0.71 ^a^	0.72 _a_
	13.7	12.3	2.4	2.4	0.2	0.2	0.05	0.03	0.00	0.00	0.01	0.01
P1	1106.1 ^a^	1090.6 _ab_	154.6 ^a^	151.7 _a_	25.9 ^a^	25.6 _a_	2.56 ^a^	2.63 _a_	0.35 ^a^	0.35 _a_	0.71 ^a^	0.72 _a_
	10.0	13.2	1.9	2.4	0.3	0.2	0.05	0.05	0.00	0.00	0.01	0.01
P2	1101.8 ^ab^	1083.0 _abc_	154.0 ^ab^	150.8 _ab_	25.8 ^ab^	25.5 _ab_	2.56 ^a^	2.59 _b_	0.35 ^a^	0.35 _a_	0.71 ^a^	0.72 _ab_
	17.8	15.0	2.9	2.4	0.4	0.4	0.05	0.05	0.00	0.00	0.01	0.01
P3	1095.2 ^b^	1078.9 _bc_	152.9 ^bc^	150.4 _ab_	25.7 ^ab^	25.3 _bc_	2.56 ^a^	2.59 _b_	0.35 ^a^	0.35 _a_	0.72 ^a^	0.72 _b_
	18.8	17.2	2.9	2.8	0.5	0.4	0.05	0.05	0.00	0.00	0.01	0.01
P4	1095.7 ^b^	1075.1 _c_	152.7 ^c^	149.8 _b_	25.8 ^b^	25.2 _c_	2.56 ^a^	2.59 _b_	0.35 ^b^	0.35 _a_	0.72 ^a^	0.72 _b_
	19.2	40.3	3.1	6.4	0.5	0.7	0.08	0.08	0.01	0.00	0.01	0.01
